# Supplementary material for: Mechanical characterization of disordered and anisotropic cellular monolayers
Source: Phys Rev E. Author manuscript; Available in PMC 2022 Jul 20. (PMC7613005; doi:10.1103/PhysRevE.97.052409)
Supplement: Appendix [file EMS146507-supplement-Appendix.pdf]

APPENDIX A: A HOMOGENEOUS SMALL-AMPLITUDE STRAIN

We derive the mappings of key geometric quantities under a small-amplitude homogeneous and symmetric strain E, which transforms position vectors as $\mathbf{R} \to \mathbf{R} + \mathsf{E}\cdot\mathbf{R}$, and then apply these mappings to the mechanical energy U.

We assume that all quantities X follow a mapping of the form $X \to X + \Delta X$, defined relative to the same cell, and therefore temporarily drop the α subscript. Tangents are defined as $\mathbf{t}^i = \mathbf{R}^{i+1} - \mathbf{R}^i$, giving $\mathbf{t}^i + \Delta\mathbf{t}^i = \mathbf{R}^{i+1} + \mathsf{E}\cdot\mathbf{R}^{i+1} - \mathbf{R}^i - \mathsf{E}\cdot\mathbf{R}^i$, and hence $\Delta\mathbf{t}^i = \mathsf{E}\cdot\mathbf{t}^i$. The length of an edge is given by $l^i = (\mathbf{t}^i\cdot\mathbf{t}^i)^{\frac{1}{2}}$. To linear order,

$$l^i + \Delta l^i = [(\mathbf{t}^i + \Delta\mathbf{t}^i)\cdot(\mathbf{t}^i + \Delta\mathbf{t}^i)]^{\frac{1}{2}} \approx [(l^i)^2 + 2\mathbf{t}^i\cdot\mathsf{E}\cdot\mathbf{t}^i]^{\frac{1}{2}}$$
$$\approx l^i(1 + \hat{\mathbf{t}}^i\cdot\mathsf{E}\cdot\hat{\mathbf{t}}^i), \tag{A1}$$

demonstrating that $\Delta l^i = l^i(\hat{\mathbf{t}}^i\cdot\mathsf{E}\cdot\hat{\mathbf{t}}^i)$. The cell perimeter is $L = \sum_{i=0}^{Z-1} l^i$, so that

$$L + \Delta L = \sum_{i=0}^{Z-1} l^i(1 + \hat{\mathbf{t}}^i\cdot\mathsf{E}\cdot\hat{\mathbf{t}}^i)$$
$$= L + \sum_{i=0}^{Z-1} l^i(\hat{\mathbf{t}}^i\cdot\mathsf{E}\cdot\hat{\mathbf{t}}^i) = L(1 + \mathsf{Q} : \mathsf{E}). \tag{A2}$$

Thus $\Delta L = L\mathsf{Q} : \mathsf{E}$. It follows that $T + \Delta T = \Gamma(L + \Delta L - L_0) = T + \Gamma L\mathsf{Q} : \mathsf{E}$. Similarly, $l^i\hat{\mathbf{t}}^i \to l^i\hat{\mathbf{t}}^i + \Delta(l^i\hat{\mathbf{t}}^i)$. From the product rule, $\Delta(l^i\hat{\mathbf{t}}^i) = (\Delta l^i)\hat{\mathbf{t}}^i + l^i(\Delta\hat{\mathbf{t}}^i)$. Now, $\Delta(l^i\hat{\mathbf{t}}^i) = l^i\mathsf{E}\cdot\hat{\mathbf{t}}^i$ and using (A1) gives $l^i\mathsf{E}\cdot\hat{\mathbf{t}}^i = l^i(\hat{\mathbf{t}}^i\cdot\mathsf{E}\cdot\hat{\mathbf{t}}^i)\hat{\mathbf{t}}^i + l^i(\Delta\hat{\mathbf{t}}^i)$. Thus, $\Delta\hat{\mathbf{t}}^i = \mathsf{E}\cdot\hat{\mathbf{t}}^i - (\hat{\mathbf{t}}^i\cdot\mathsf{E}\cdot\hat{\mathbf{t}}^i)\hat{\mathbf{t}}^i$. Note that $\hat{\mathbf{t}}^i\cdot(\Delta\hat{\mathbf{t}}^i) = 0$, ensuring that unit vectors are rotated but not stretched.

We now consider the deformation $L\mathsf{Q} \to L\mathsf{Q} + \Delta(L\mathsf{Q})$, writing

$$L\mathsf{Q} + \Delta(L\mathsf{Q}) = \sum_{i=0}^{Z-1}[l^i\hat{\mathbf{t}}^i + \Delta(l^i\hat{\mathbf{t}}^i)][\hat{\mathbf{t}}^i + \Delta\hat{\mathbf{t}}^i]$$
$$= \sum_{i=0}^{Z-1}[l^i\hat{\mathbf{t}}^i + l^i\mathsf{E}\cdot\hat{\mathbf{t}}^i] \times [\hat{\mathbf{t}}^i + \mathsf{E}\cdot\hat{\mathbf{t}}^i - (\hat{\mathbf{t}}^i\cdot\mathsf{E}\cdot\hat{\mathbf{t}}^i)\hat{\mathbf{t}}^i]$$
$$\approx \sum_{i=0}^{Z-1} l^i\hat{\mathbf{t}}^i\hat{\mathbf{t}}^i + \sum_{i=0}^{Z-1}\{l^i\hat{\mathbf{t}}^i[\mathsf{E}\cdot\hat{\mathbf{t}}^i - (\hat{\mathbf{t}}^i\cdot\mathsf{E}\cdot\hat{\mathbf{t}}^i)\hat{\mathbf{t}}^i] + l^i(\mathsf{E}\cdot\hat{\mathbf{t}}^i)\hat{\mathbf{t}}^i\} \tag{A3}$$

to linear order. Thus we see that $\Delta(L\mathsf{Q}) = L\mathsf{B} : \mathsf{E}$, where B in component form is

$$\{\mathsf{B}_\alpha\}_{pqrs} = \frac{1}{L_\alpha}\sum_{i=0}^{Z_\alpha-1} l^i_\alpha\Bigg[\frac{1}{2}\big(\hat{t}^i_{\alpha,p}\mathsf{I}_{qr}\hat{t}^i_{\alpha,s} + \hat{t}^i_{\alpha,q}\mathsf{I}_{pr}\hat{t}^i_{\alpha,s} + \hat{t}^i_{\alpha,p}\mathsf{I}_{qs}\hat{t}^i_{\alpha,r} + \hat{t}^i_{\alpha,q}\mathsf{I}_{ps}\hat{t}^i_{\alpha,r}\big) - \hat{t}^i_{\alpha,p}\hat{t}^i_{\alpha,q}\hat{t}^i_{\alpha,r}\hat{t}^i_{\alpha,s}\Bigg], \tag{A4}$$

ensuring that $\{\mathsf{B}_\alpha\}_{pqrs} = \{\mathsf{B}_\alpha\}_{qprs} = \{\mathsf{B}_\alpha\}_{pqsr}$.

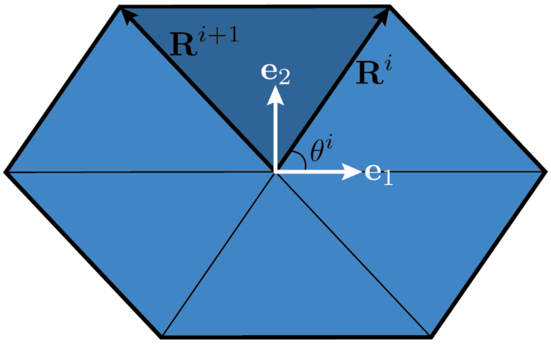

FIG. 8. Representative geometry used to calculate the area of a cell.

To evaluate area changes, we begin by considering the area of a subtriangle, $\triangle = \{\mathbf{R}_\alpha, \mathbf{R}^i_\alpha, \mathbf{R}^{i+1}_\alpha\}$, comprising the cell centroid and two vertices from an edge (Fig. 8). We have used the α subscript notation for clarity in defining the centroid, but drop it again from this point. The area of this triangle is $A^\triangle = \frac{1}{2}\hat{\mathbf{z}}\cdot(\mathbf{R}^i \times \mathbf{R}^{i+1})$. Applying E, we have

$$A^\triangle + \Delta A^\triangle = \tfrac{1}{2}\hat{\mathbf{z}}\cdot(\mathbf{R}^i + \mathsf{E}\cdot\mathbf{R}^i)\times(\mathbf{R}^{i+1} + \mathsf{E}\cdot\mathbf{R}^{i+1})$$
$$\approx \tfrac{1}{2}\hat{\mathbf{z}}\cdot(\mathbf{R}^i\times\mathbf{R}^{i+1} + \mathbf{R}^i\times\mathsf{E}\cdot\mathbf{R}^{i+1} + \mathsf{E}\cdot\mathbf{R}^i\times\mathbf{R}^{i+1}) \tag{A5}$$

to linear order. Since E is symmetric, we can make use of the spectral theorem to write $\mathsf{E} = \lambda_1\mathbf{e}_1\mathbf{e}_1 + \lambda_2\mathbf{e}_2\mathbf{e}_2$, where $(\mathbf{e}_1,\mathbf{e}_2)$ form an orthonormal basis of eigenvectors. Inserting into (A5) we have

$$\Delta A^\triangle = \tfrac{1}{2}\hat{\mathbf{z}}\cdot[\lambda_1\mathbf{R}^i\times\mathbf{e}_1\mathbf{e}_1\cdot\mathbf{R}^{i+1} + \lambda_2\mathbf{R}^i\times\mathbf{e}_2\mathbf{e}_2\cdot\mathbf{R}^{i+1} + \lambda_1(\mathbf{e}_1\mathbf{e}_1\cdot\mathbf{R}^i)\times\mathbf{R}^{i+1} + \lambda_2(\mathbf{e}_2\mathbf{e}_2\cdot\mathbf{R}^i)\times\mathbf{R}^{i+1}]. \tag{A6}$$

Defining θ^i and θ^{i+1} such that $\lambda_1\mathbf{R}^i\times\mathbf{e}_1 = -\lambda_1|\mathbf{R}^i|\sin\theta^i\hat{\mathbf{z}}$, and $\mathbf{e}_1\cdot\mathbf{R}^{i+1} = |\mathbf{R}^{i+1}|\cos\theta^{i+1}$ (see Fig. 8) gives

$$\Delta A^\triangle = \frac{1}{2}|\mathbf{R}^i||\mathbf{R}^{i+1}|\Bigg[-\lambda_1\sin\theta^i\cos\theta^{i+1} + \lambda_2\sin\left(\frac{\pi}{2}-\theta^i\right)\cos\left(\frac{\pi}{2}-\theta^{i+1}\right) + \lambda_1\cos\theta^i\sin\theta^{i+1} - \lambda_2\cos\left(\frac{\pi}{2}-\theta^i\right)\times\sin\left(\frac{\pi}{2}-\theta^{i+1}\right)\Bigg]$$
$$= \frac{1}{2}|\mathbf{R}^i||\mathbf{R}^{i+1}|[\lambda_1\sin(\theta^{i+1}-\theta^i) + \lambda_2\sin(\theta^{i+1}-\theta^i)] = \mathrm{Tr}(\mathsf{E})A^\triangle. \tag{A7}$$

Summing over subtriangles gives $\Delta A = \mathrm{Tr}(\mathsf{E})A$.

Having shown that under the deformation $\mathbf{R} \to \mathbf{R} + \mathsf{E}\cdot\mathbf{R}$, lengths and areas of individual cells transform according to $L_\alpha \to L_\alpha(1 + \mathsf{Q}_\alpha : \mathsf{E})$, $A_\alpha \to A_\alpha(1 + \mathrm{Tr}(\mathsf{E}))$, it is straightforward to determine the associated change in recoverable

mechanical energy (3) as

$$\Delta U = \sum_\alpha \Delta U_\alpha = \sum_\alpha P_\alpha \Delta A_\alpha + T_\alpha \Delta L_\alpha$$
$$= \sum_\alpha (P_\alpha A_\alpha \mathsf{I} + T_\alpha L_\alpha \mathsf{Q}_\alpha) : \mathsf{E} = \mathbf{\Sigma}^{(e)} : \mathsf{E}, \quad \text{(A8)}$$

where $\mathbf{\Sigma}^{(e)} \equiv -\sum A_\alpha \boldsymbol{\sigma}_\alpha^{(e)}$. Here, we have decomposed the stress in (9) into its elastic and viscous components $\boldsymbol{\sigma}_\alpha = \boldsymbol{\sigma}_\alpha^{(e)} + \boldsymbol{\sigma}_\alpha^{(v)}$. The sign change between $\boldsymbol{\sigma}$ and $\mathbf{\Sigma}$ arises from the difference between the stresses exerted on, or by, a cell or tissue. Similarly defining $\mathbf{\Sigma}^{(v)} \equiv -\sum A_\alpha \boldsymbol{\sigma}_\alpha^{(v)}$, and noting that for this small deformation $\dot{A}_\alpha = A_\alpha \mathrm{Tr}(\dot{\mathsf{E}})$, it follows that

$$\mathbf{\Sigma}^{(v)} : \dot{\mathsf{E}} = \sum_\alpha \gamma A_\alpha \dot{A}_\alpha \mathrm{Tr}(\dot{\mathsf{E}}) + \mu L_\alpha \dot{L}_\alpha \mathsf{Q}_\alpha : \dot{\mathsf{E}}$$
$$= \sum \gamma \dot{A}_\alpha^2 + \mu \dot{L}_\alpha^2 = \Phi, \quad \text{(A9)}$$

where $\Phi = \sum_\alpha \Phi_\alpha$ is the dissipation rate (5). In the absence of neighbor exchanges, which would contribute additional stresses and deformations (treated in a coarse-grained approximation by Ishihara *et al.* [10]), the total rate of change of internal energy of the system is therefore $\dot{\mathcal{E}} = \mathbf{\Sigma} : \dot{\mathsf{E}} = (\mathbf{\Sigma}^{(e)} + \mathbf{\Sigma}^{(v)}) : \dot{\mathsf{E}} = \dot{U} + \Phi = 0$ [by (6)]. Positing the thermodynamic relation $\Delta\mathcal{E} = T\Delta S + \Delta U$, where ΔS is an entropy change at temperature T, it follows that for the imposed deformation E, $T\dot{S} = -\dot{U} = \Phi \geqslant 0$.

APPENDIX B: EXPERIMENTAL METHODS

Xenopus laevis female frogs were preprimed 4–7 days in advance with 50 units of pregnant mare's serum gonadotrophin (Intervet UK) and then primed with 500 units of human chorionic gonadotrophin (Intervet UK) 18 h before use as detailed in Ref. [50]. Each frog was housed individually overnight and transferred to room-temperature1× Marc's modified Ringer's (MMR) solution [100 mM NaCl, 2 mM KCl, 1 mM MgCl, and 5 mM 4-(2-hydroxyethyl)-1-piperazine-ethanesulfonic acid (HEPES) (*p*H 7.4)] at least 2 h prior to egg collection. *In vitro* fertilization was performed as described previously [50] and embryos were dejellied using 2% cysteine (in 0.1× MMR, *p*H 7.8–8.0). Embryos were microinjected with a needle volume of 5 and 2.5 nl at the two- or four-cell stages respectively, into all cells, using a Picospritzer III (Parker instrumentation) with embryos submerged in 0.1× MMR plus 5% Ficoll. RNA was synthesized as described previously [51] and microinjected at the following needle concentrations: 0.5 mg/ml GFP-α-tubulin; 0.1 mg/ml cherry-histone2B [52].

Animal cap tissue was dissected from the embryo at stage 10 of development (early gastrula stage) following a protocol previously described by Joshi and Davidson [53], and cultured on a 20 mm × 20 mm × 1 mm elastomeric PDMS membrane coated with fibronectin (incubated at 4 °C overnight with 1 ml of 10 μg/ml fibronectin). The fibronectin was removed and each membrane was subsequently washed three times with 1× phosphate buffered saline (PBS) followed by two washes with Danilchik's for Amy explant culture media (DFA; 53 mM $NaCl_2$, 5 mM Na_2CO_3, 4.5 mM potassium gluconate, 32 mM sodium gluconate, 1 mM $CaCl_2$, 1 mM $MgSO_4$) prior to the introduction of each animal cap. Animal cap explants were excised using forceps and hair knives to make neat squares of tissue. Each explant was transferred to a PDMS membrane filled with DFA and a coverslip with vacuum grease at each end was placed over the top to ensure the explant adhered to the fibronectin-coated membrane. Each membrane was then incubated at 18 °C for at least 2 h prior to imaging.

Each PDMS membrane was attached to a stretch apparatus (custom made by Deben UK Limited) fixed securely to the stage of a Leica TCS SP5 acousto-optical beam splitter (AOBS) upright confocal and a 0.5 or 8.6 mm uniaxial stretch was applied for control (unstretched) and stretched samples, respectively. Images were collected on the Leica TCS SP5 AOBS upright confocal using a 20×/0.50 HCX Apo U-V-I [W (dipping lens)] objective and 2× (or 1×) confocal zoom. The confocal settings were as follows: pinhole 1 Airy unit, scan speed 400 Hz bidirectional, format 512 × 512 (or 1024 × 1024). Images were collected using hybrid detectors with the following detection mirror settings: HyD2 fluorescein isothiocyanate (FITC) 500–550 nm; HyD4 Texas red 590–690 nm using the 488 nm (20%) and 543 nm (100%) laser lines, respectively. Images were collected sequentially. The distance between each optical stack was maintained at 4.99 μm and the time interval between each capture was restricted to 20 s, with each sample being imaged for up to 2.5 h. Only the maximum intensity projections of these 3D stacks are shown in the results.

APPENDIX C: ORDER PARAMETER

A more traditional measure of spatial disorder is provided by the order parameter $Q = \langle \cos 2\theta \rangle$, where θ measures the angle of the principal axis of the shape tensor of each cell ($\sum_{i=0}^{Z_\alpha - 1} \mathbf{R}_\alpha^i \otimes \mathbf{R}_\alpha^i$) with respect to the direction of stretch and the average is taken over all the cells in the monolayer. Figure 9 illustrates the evolution of Q for the stretch realizations illustrated in Fig. 4(b). It is notable that while the two examples are quite similar by this geometric measure, the shear stress [Fig. 4(b)] is substantially lower for parameters closer to the region I/II boundary in parameter space [Fig. 1(b)].

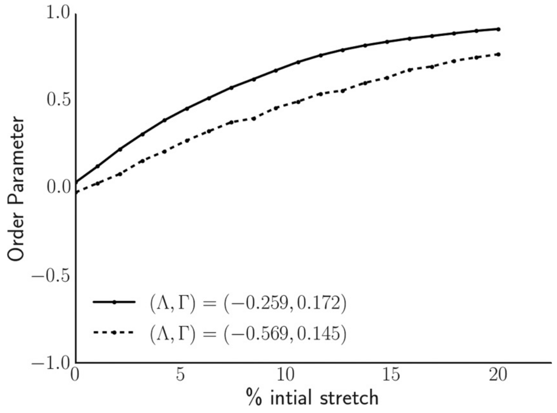

FIG. 9. The order parameter Q, for the realizations plotted in Fig. 4(b).
